# Nano-hybrid plasmonic photocatalyst for hydrogen production at 20% efficiency

**DOI:** 10.1038/s41598-017-09261-7

**Published:** 2017-08-17

**Authors:** Mariia V. Pavliuk, Arthur B. Fernandes, Mohamed Abdellah, Daniel L. A. Fernandes, Caroline O. Machado, Igor Rocha, Yocefu Hattori, Cristina Paun, Erick L. Bastos, Jacinto Sá

**Affiliations:** 1 Departament of Chemistry, Ångström Laboratory, Uppsala University, 75120 Uppsala, Sweden; 2 Departament of Fundamental Chemistry, Institute of Chemistry, University of São Paulo, 05508-000 São Paulo, Brazil; 3 Department of Chemistry, Qena Faculty of Science, South Valley University, 83523 Qena, Egypt; 4 Departament of Engineering Sciences, Ångström Laboratory, Uppsala University, 75121 Uppsala, Sweden; 5 Institute of Physical Chemistry, Polish Academy of Sciences, 01-224 Warsaw, Poland

## Abstract

The efficient conversion of light energy into chemical energy is key for sustainable human development. Several photocatalytic systems based on photovoltaic electrolysis have been used to produce hydrogen via water reduction. However, in such devices, light harvesting and proton reduction are carried separately, showing quantum efficiency of about 10–12%. Here, we report a nano-hybrid photocatalytic assembly that enables concomitant reductive hydrogen production and pollutant oxidation with solar-to-fuel efficiencies up to 20%. The modular architecture of this plasmonic material allows the fine-tuning of its photocatalytic properties by simple manipulation of a reduced number of basic components.

## Introduction

Artificial conversion of solar light into chemical bonds (*aka* artificial photosynthesis) has attracted wide interest because it yields storable energy deprived of greenhouse gas emission^1, 2^. The development of catalysts for photoinduced electron/hole transfer allows the production of molecular hydrogen and oxygen gases from water reduction and oxidation, respectively^3^. However, oxygen production relies on a difficult to achieve multi-step four-electron process, which yields a product with low-economic value, difficult to separate, and a direct competitor of protons for the photo-generated electrons. Strides have been made in the mitigation of the later problems by separating H_2_ and O_2_ evolution in space^4^ and time^5^ but fewer efforts are been done seeking for alternative substrates (e.g. pollutants), which can improve process safety and economic viability^6^. Moreover, H_2_ usages go beyond fuel applications^7, 8^, and include the synthesis of several bulk and fine chemicals, *e.g*. ammonia, methanol, fragrances and drugs. Depending on the approach, massive hydrogen production via water splitting may increase the concentration of oxygen in the atmosphere, an undesirable event that can affect profoundly the planet, as it did *ca*. 2.4 billion years ago^9, 10^.

Low-cost devices for the large-scale production of solar fuels can mitigate the use of fossil fuels. Developments based on large band gap semiconductors (>3 eV), such as TiO_2_^3^, are hindered by the need of UV irradiation to induce charge separation. Efforts to shift their absorption to the visible range resulted in the decrease of both reaction scope and overall performance^11^. A promising strategy is semiconductor sensitization, as the case of dye-sensitized solar cells (DSSCs) concept^12^, where the sensitizer harvests sunlight and rapidly injects electrons (τ_inj_ < 1 ps) into the TiO_2_ conduction band (CB). Metallic nanoparticles (NPs) are attractive sensitizers due to their large optical cross-sections related to the excitation of localized surface plasmons (LSP). Gold group metals exhibit LSP resonances in the visible region, and their absorption maxima depend on nanoparticle morphology, enabling a good match with the solar spectrum. Additionally, their *d* ^10^ configuration bestows them chemical stability. Recently, the excitation of Au and Ag LSP resonances were shown to improve charge transfer in DSSCs^13^, photocurrents^14^, and photocatalytic oxidations^15, 16^.

Hallett-Tapley and coauthors^17^ suggested three LSP mediated reactive processes: thermal, electronic or antenna. The electronic process is the relevant for light conversion to chemical bonds, inferring hot electron creation^18^. Their generation upon LSP excitation was recently established via Au L_III_-edge high-resolution X-ray absorption spectroscopy^19^. LSP excitation led to an upward shift in the threshold ionization energy (*ca*. 1.0 eV), and an increase of Au *d*-band hole population, consistent with hot electrons formation. The lifetime of hot electrons is too short (<100 fs) for any catalytic reaction to happen. However, transfer of hot electrons to the CB of TiO_2_ prolongs their lifetime to microseconds^19^, as confirmed by transient mid-IR spectroscopy^20^.

Another challenge to produce efficient solar fuels is the conciliation of light-induced single electron transfer with multi-electron catalysis. Systems must be able to accumulate/store charge particles provided by the fast light absorption process so the slow multi-electron catalysis can take place, i.e. *accumulative charge separation concept* ^21^. Metal NPs contain large amount of loosely bonded electrons that, combined with the capacity of LSP to redirect the light flow towards the NP (i.e. *Poynting vector*), makes multi-electron generation per single NP plausible. Small metal NPs are excellent hydrogen evolution catalysts, allowing charge accumulation and storage^22^. Gold NPs fabricated using *top-down* approaches have been extensively used for LSP-based conversion and storage of solar energy^23^. However, the bottom-up synthesis in solution of alternative silver NPs photocatalytic systems has been pursued both for practical and economic reasons.

Here, we present a modular nano-hybrid assembly fabricated using silver NPs produced via *bottom-up* synthesis. This chemical system is made up of a discrete number of components connected via covalent bonding and intermolecular interactions. In the present case, the subunits are silver metallic nanoparticles and *n*-type semiconductor TiO_2_ nanoparticles interconnected via a molecular linker. Our approach allows for the synthesis of a multitude of multifuctional materials from a relatively small number of basic units. The nano-hybrid architectures can accomplish long-lived charge separate states, enabling concomitant photocatalytic production of H_2_ and pollutant oxidation.

## Results and Discussion

**Figure 1:**
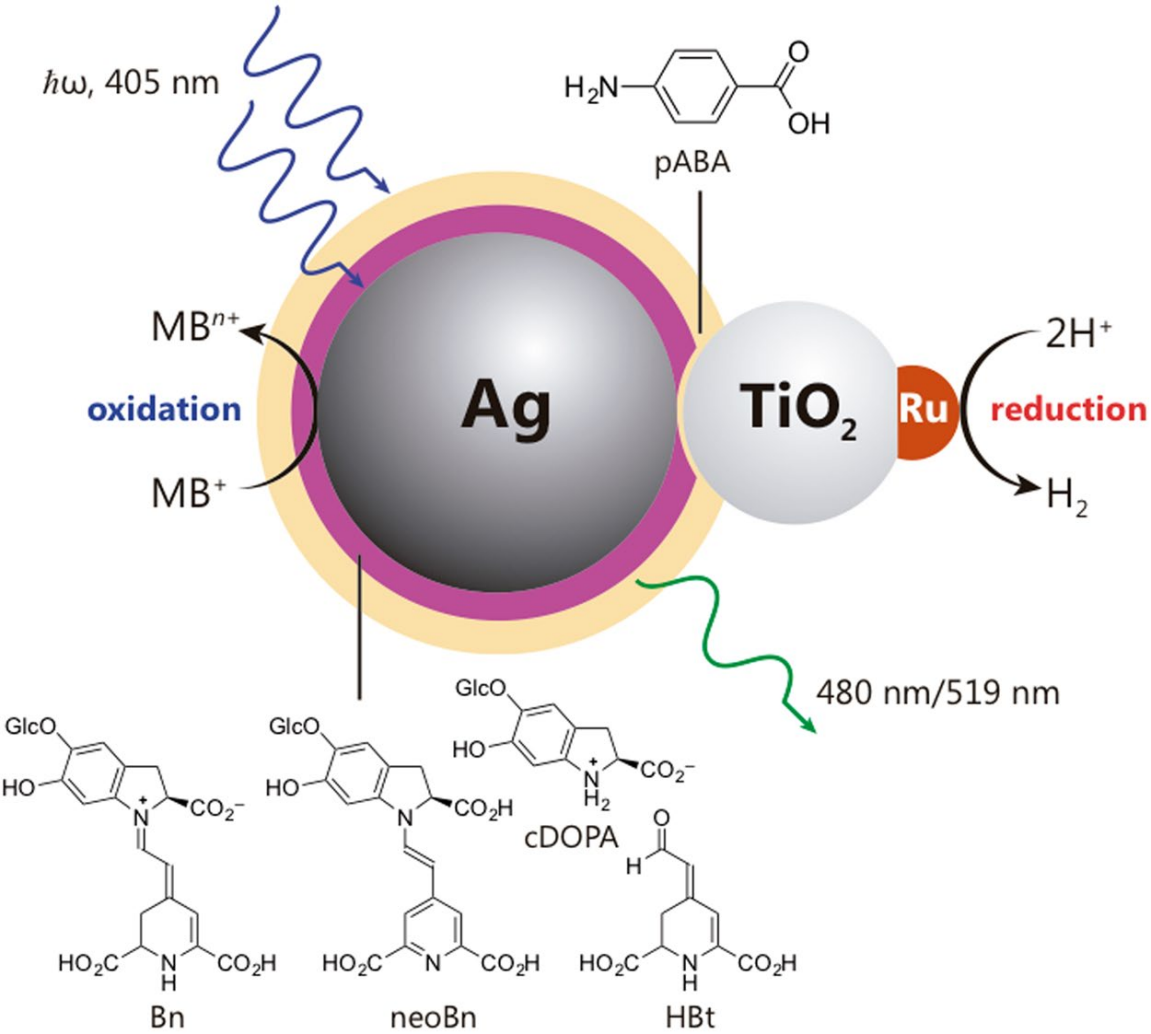
Nano-hybrid assembly synthesized using modular design approach. The architecture is comprised of light absorber and oxidation catalyst (Ag NPs), a molecular wire linker (pABA), a semiconductor (TiO_2_), a co-catalyst (Ru NPs) and regenerators (Bts).

Figure 1 shows the blueprint of the nano-hybrid system, composed by natural product-stabilized Ag NPs, a molecular wire linker, TiO_2_ as a semiconductor and Ru NPs as a co-catalyst. Silver NPs stabilized with betanin and derivatives (Bts-Ag NPs) were synthesized in an automated microfluidic reactor using pure betanin extracted from beetroots^24^, as reducing and capping agent. A genetic optimization algorithm was used to ensure that particles size and light absorption are homogeneous^25^. Herein, we used monodispersed spherical Bts-Ag NPs with 28–30 nm, exhibiting a LSP resonance absorption maximum wavelength (λ_max_) at 405 nm; refer to the supporting information (SI) for DLS analysis (Fig. S2), SEM (Fig. S3), UV-Vis spectrum (Fig. S4), and AFM (Fig. S5).

Betanin (Bn) and its derivatives from alkaline hydrolysis or oxidation, betalamic acid (HBt), cyclo-DOPA 5-*O*-glucoside (cDOPA), and neobetanin (neoBn), are expected to retard electron/hole recombination and are used as *regenerators*. TiO_2_ semiconductor was used to enhance electron lifetime^19^ and ruthenium NPs as hydrogen evolution catalyst^26^, which were prepared via chemical reduction (see SI).

A molecular linker (4-aminobenzoic acid, pABA) was used to enhance the electronic coupling between the electron donor (Ag NPs) and the electron acceptor (TiO_2_). Electron transfer through a *π*-conjugated molecular wire is essential to a multitude of biologic systems and molecular electronics^27^ but has seldom usage in the synthesis of nano-based artificial photosynthesis systems. The linker pABA provides an excellent electronic coupling because of its good *π*-conjugated, rigidity and planarity^28^. The connectivity between electron donor and acceptor parts via molecular linker was accessed via Raman spectroscopy (Fig. S6). Due to the similarities in functional groups between betanin derivatives and pABA, there are not many characteristic functionalities that can be firmly used for the assignment. There is, however, a peak at 1727 cm^−1^ in the silver containing samples that is absent in the pure linker, assigned to C=O terminal group of betalamic acid^29^. Addition of the linker to Bts-Ag NPs led to the appearance of a small peak at 1605 cm^−1^, ascribed to secondary amine N–H bend, a broad peak from 1620 to 1800 cm^−1^, associated to aromatic ring combination band (1660–2000 cm^−1^) and carboxylic acid (1700–1725 cm^−1^)^29^, suggesting the anchoring of the amino group of the linker to the Bts-Ag NPs. Addition of TiO_2_-Ru led to a decrease in the ratio between the broad peak and 1605 cm^−1^, suggesting the anchoring of the carboxylic group of the linker to TiO_2_.

**Figure 2:**
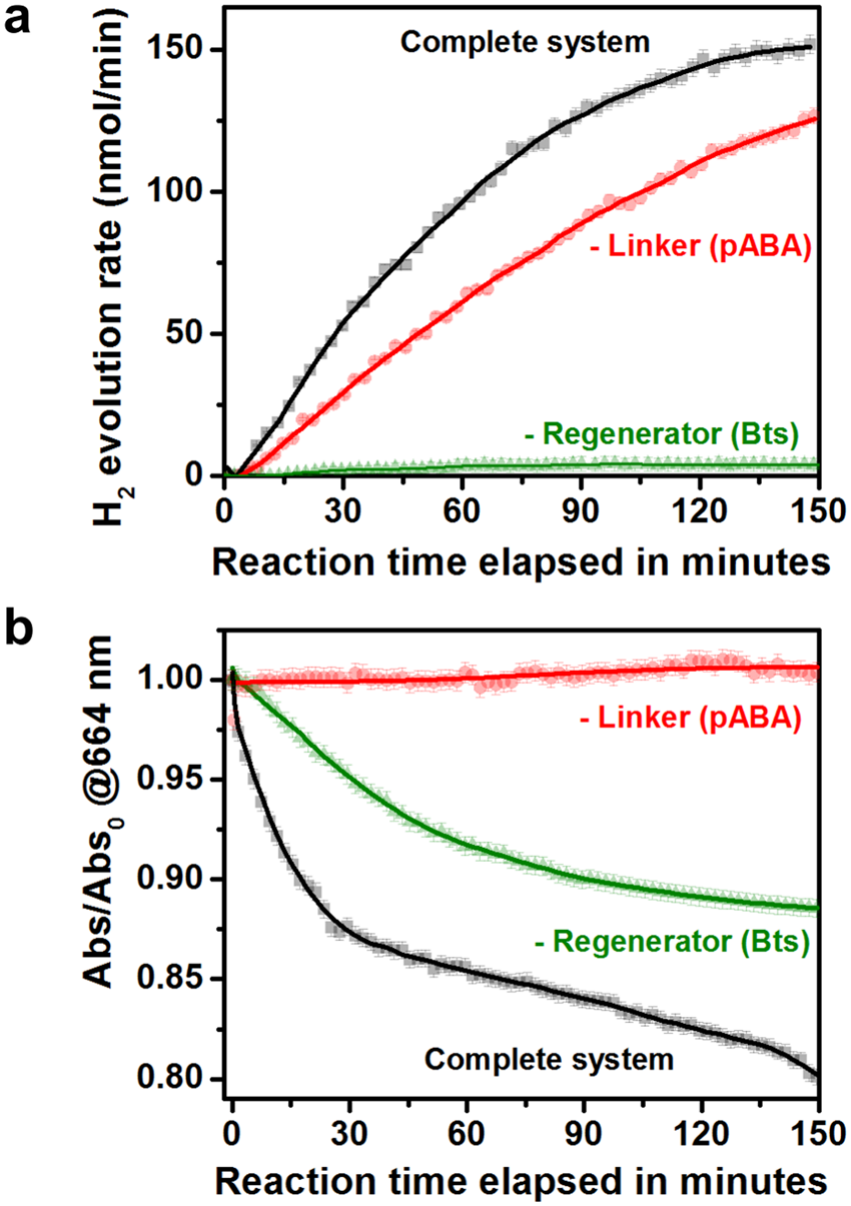
Effect of components in hydrogen production and methylene blue (MB^+^) oxidation. (**a**) Hydrogen production and (**b**) methylene blue (MB^+^) oxidation from the complete silver based nano-hybrid assembly (Bts-Ag NP–linker–TiO_2_–Ru NPs), without the regeneration layer (–Regenerator (Bts)) and without the molecular wire linker (–pABA), upon monochromatic excitation at 405 nm.

Both the molecular wire linker and the regenerator are important for photocatalytic hydrogen production (Fig. 2a) and methylene blue (model pollutant) depletion (Fig. 2b). Experiments were carried out using a flow photoreactor (Fig. S1)^30^. The results show a noteworthy increase in hydrogen evolution when the systems are connected by a molecular wire. Such increase was not observed using PVP-Ag NPs, prepared in the absence of betanin via the polyol method (Fig. 2a) confirming the active role of the capping layer in the process, as rationalized below. Transient absorption infrared spectroscopy measurements performed on complete system with PVP-Ag NPs, revealed very fast charge recombination, which justifies the low activity.

A noticeable rise in the multi-electron photo-oxidation of methylene blue was detected for the complete system, contrasting with the low activity of the unconnected system (Fig. 2b) in which electron transfer occurs via random collision between Bts-Ag NPs (electron donor) and TiO_2_:Ru NPs (electron acceptor). Individual components show no photocatalytic activity (control experiments are shown in Fig. S7). There is however evidences for methylene blue adsorption on the control experiment using only TiO_2_:Ru NPs, reflected in absorption signal drop and large oscillation in the date, which is to be expected due to TiO_2_ large surface area. Preliminary tests show that the complete system is stable for at least 5 h on stream and recyclable, since the second run of photocatalytic test show virtually no change in photoactivity. Raman and AFM measurements performed after the photocatalytic tests revealed no significant differences between fresh, used in the photocatalytic tests and aged for 3 weeks samples.

The solar-to-fuel efficiency (quantum yield) of the nano-hybrid system was measured directly, assuming the steady-state amount of H_2_ produced (150 nmol min^−1^) at a known photon flux. A quantum yield of 19.9 ± 0.5% was measured assuming that every photon absorbed has an equal probability of generating a hot electron and a reaction stoichiometry of two electrons for each H_2_ molecule formed. Currently, the best performing systems based on photovoltaic electrolysis reach 10–12% conversion efficiency. For example, Luo and coworkers^31^ reported recently 12.3% water photolysis efficiency via perovskite photovoltaics and earth-abundant catalysts. Jacobsson and collaborators^32^ described a monolithic water splitting device based on series interconnected thin film absorbers capable of attaining 10% solar-to-hydrogen efficiency. Kim and coworkers^33^ developed a dual photoanodes-silicon solar cell, reaching an unbiased water splitting efficiency of 7.7%. Finally, Nocera’s artificial leaf model without precious metals water-splitting catalysts can attain a 10% solar-to-fuels efficiency^34^. Apart of superseding the current efficiencies, the reported value was obtained without component ratio optimization, leaving large room for further improvement.

**Figure 3:**
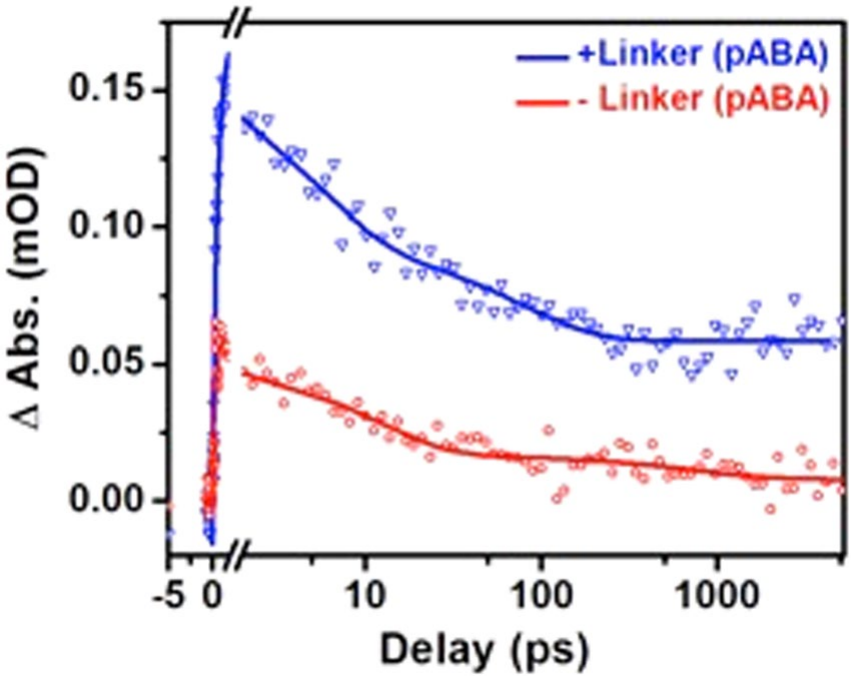
Effect of linking Ag NPs to TiO_2_ with a molecular linker to the ultrafast transient infrared absorption signal. Kinetic traces extracted at 2081 cm^−1^ from transient absorption infrared spectroscopy measurements upon excitation at 405 nm for the Bts-Ag NP–linker–TiO_2_ (linked system, + linker (pABA)) and Bts-Ag NP–TiO_2_ (unlinked system, – linker (pABA)).

The mechanism of electron transport from donor to acceptor site by the molecular wire linker was investigated by performing ultrafast transient infrared absorption measurements. Infrared spectroscopy is a very sensitive technique to evaluate the arrival of electrons to TiO_2_ conduction band because photoinduced charge carriers in semiconductor conduction band have mid-IR absorption originated from the quasi-metallic state formed^20^. Figure 3 shows the kinetic trace extracted at 2081 cm^−1^ from transient absorption infrared spectroscopy measurements upon excitation at 405 nm for the Bts-Ag NP–linker–TiO_2_ (linked system) and Bts-Ag NP–TiO_2_ (unlinked system). Analysis of signal intensity shows that the presence of the organic linker increased by roughly 3-4-fold the transference of electrons from Ag NPs to the TiO_2_ conduction band comparatively to the unlinked system, which relies on collisional electron transfer. Kinetic fitting of the transient signal revealed a rising edge time component of ca. 600–700 fs that is significantly higher than the instrument response function (*ca*. 200 fs), assigned to the time necessary for electron transfer from Bts-Ag NPs to TiO_2_. The signal was fitted with two exponential decay functions with τ_1_ = 5 ps (93%) and τ_2_ = 80 ps (7%) (<τ> = 10 ps), which endorses ultrafast recombination dynamics and accordingly good connectivity between two sites. Note that roughly 30% of the initial signal is still present after 5 ns, our longest time delay line, suggesting electron stabilization and consequent increase in charge separation, necessary for photocatalysis to occur.

**Figure 4:**
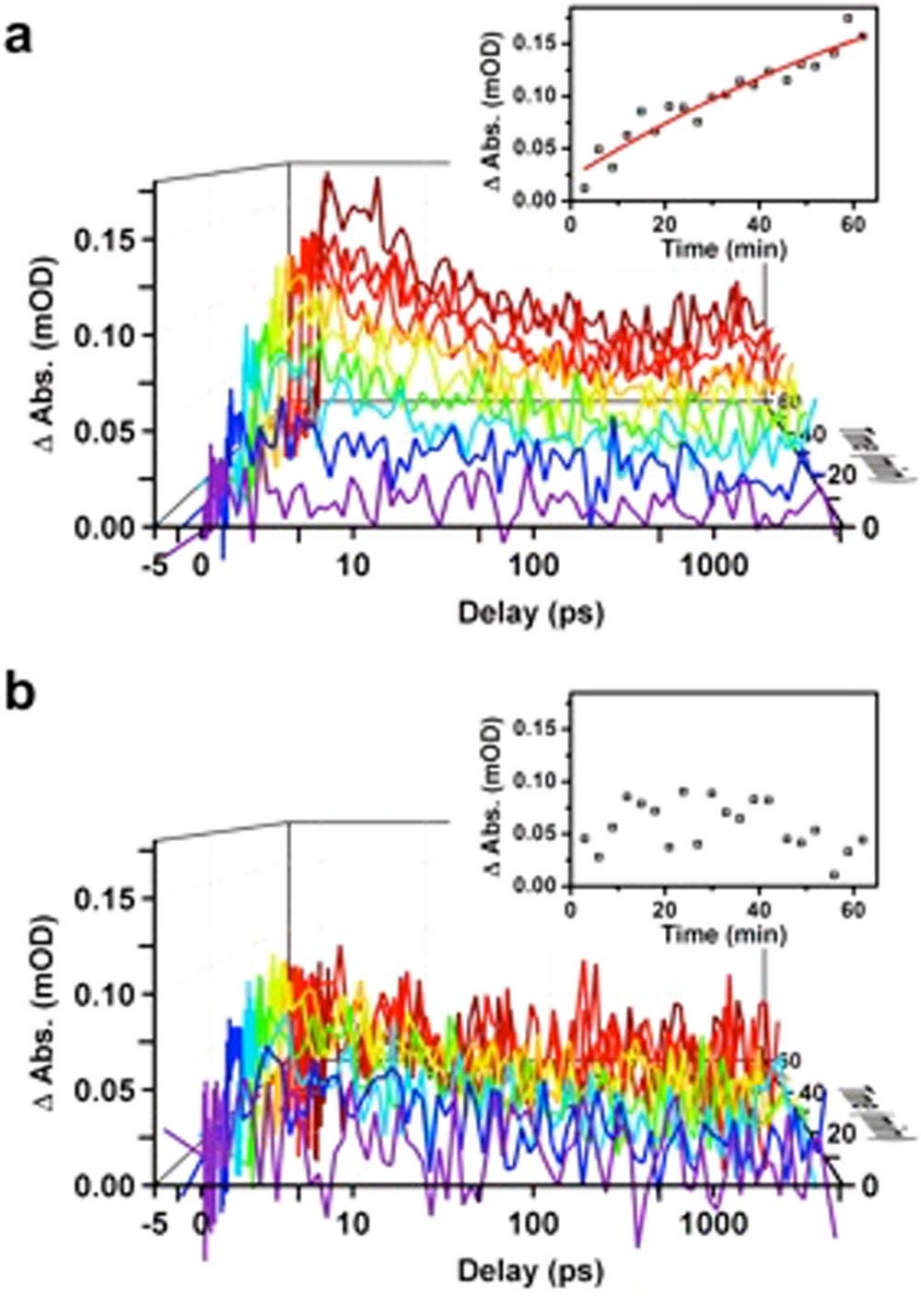
Evolution of ultrafast transient infrared absorption signal as Ag NPs connect to TiO_2_ with a molecular linker. Temporal evolution of the kinetic traces extracted at 2081 cm^−1^ from transient absorption infrared spectroscopy measurements upon excitation at 405 nm for the (**a**) Bts-Ag NP–linker–TiO_2_ (linked system), and (**b**) Bts-Ag NP–TiO_2_ (unlinked system). Inserts show the infrared absorption intensity over time extracted at 1.5 ps.

The measurement was repeated during the self-assembling of the nano-hybrid system and control experiments were carried out using unlinked nanoparticles, i.e. in the absence of the molecular linker pABA. The intensity of the transient absorption infrared spectroscopy signal increases with time when in the presence of the molecular linker, but remains unchanged for the unlinked system (Fig. 4a,b). This is particularly noticeable in the inserts, where infrared intensity extracted upon completion of charge transfer (1.5 ps) is plotted over time. There is a clear increase in intensity until the process of linking donor and acceptor is completed, which takes up to 90 min after joining all the components. Again, there is virtually no signal change in the absence of pABA (Fig. 4b and insert).

**Figure 5:**
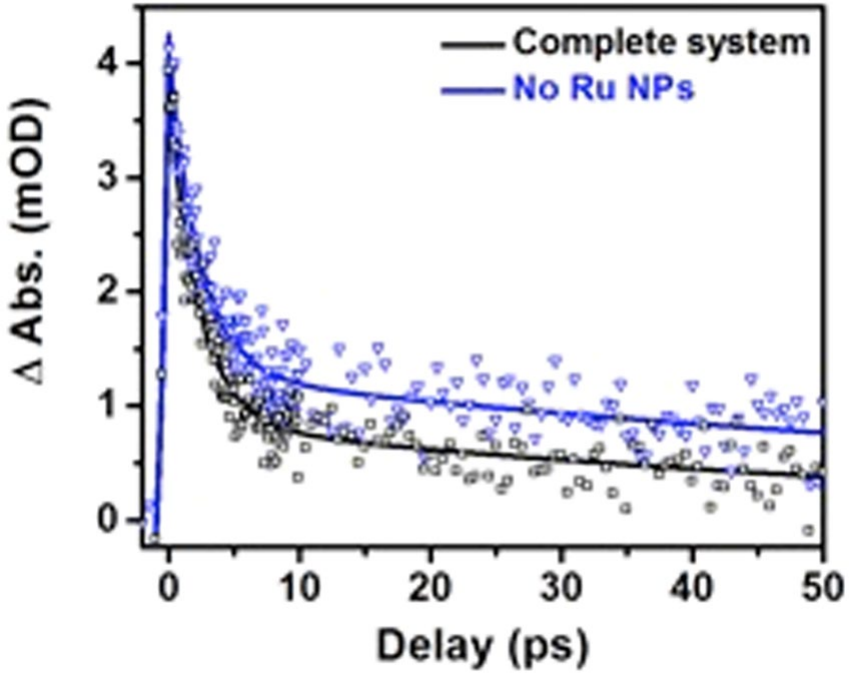
Effect of adding the catalyst to the ultrafast transient absorption signal. Kinetic traces extracted at 795 nm from transient absorption spectroscopy measurements upon excitation at 405 nm for the complete system and in the absence of Ru NPs (hydrog en evolution catalyst). The instrument response function is *ca*. 200 fs.

Ultrafast transient infrared absorption measurements show that the molecular linker improve the electronic coupling between electron donor and electron acceptor units of the nano-hybrid assembly. However, electrons must be trapped at the surface of TiO_2_ to be transferred to Ru NPs and conversely participate in a photocatalytic reaction. Ultrafast transient infrared absorption usually does not allow for distinction of bulk and surface-trapped electrons due to characteristic narrow spectral range of laboratory setups. However, surface-trapped electrons on TiO_2_ yield an absorption band at 795 nm^35^ that can be monitored by ultrafast transient absorption measurements (Fig. 5). Experiments were carried out either using the complete nano-hybrid system or in the absence of Ru NPs. In both cases, the signal has a rising edge time component of ca. 800 fs (without Ru NPs 800 fs, and complete system 820 fs) that was assigned to the time necessary for the electron transfer from Bts-Ag NPs to TiO_2_ and filling of its surface trap states. Since the rising edge component only differs by roughly 100–200 fs from the one extracted from transient infrared measurements, one can conclude that electrons fill the surface trap states rather quickly.

Conversely, good connectivity between the two sites should result in an intensification of the undesired recombination path, characterized by decays in the ultrafast domain. The signal was fitted with two exponential decay functions with τ_1_ = 2.6 ps (65%) and τ_2_ = 92 ps (35%) (<τ>  = 34 ps), which endorses ultrafast recombination dynamics and accordingly good connectivity between two sites. By adding Ru NPs one expects a suppression of the recombination pathway as electrons are expected to transfer from TiO_2_ to the Ru *4d* empty states, leading to an increase of the charge separation state lifetime. Transient absorption measurements showed faster decay when Ru NPs were present (τ_1_ = 2.6 ps (75%) and τ_2_ = 67 ps (25%), (<τ> = 19 ps)) upholding the suggestion that hot electrons are transferred to the Ru NPs after reaching TiO_2_ CB.

**Figure 6:**
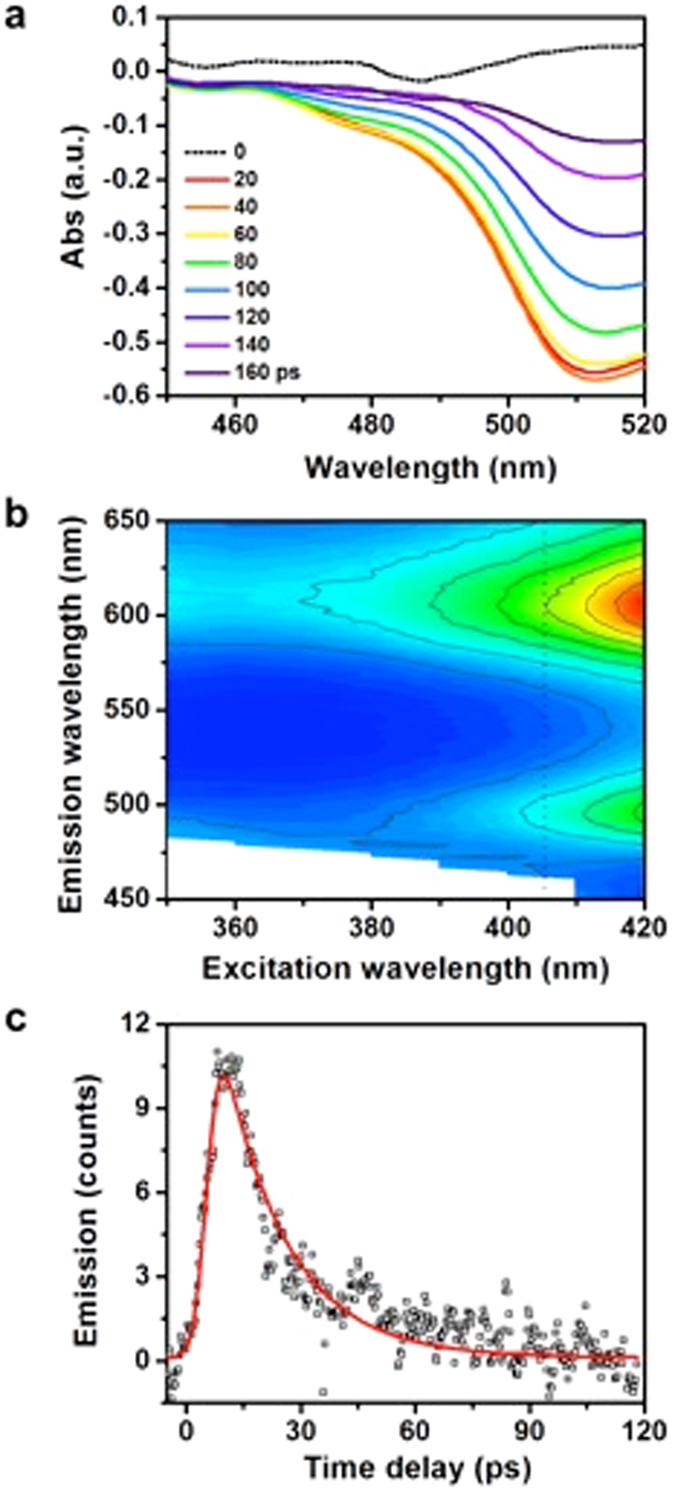
Photoluminescence signal emanated from Ag NPs regenerating layer. (**a**) Steady-state photoluminescence emanated from the complete system during photocatalysis. (**b**) Steady-state fluorescence profile of betanin in water (10 ppm AcOEt) at pH = 12. (**c**) Kinetic trace extracted at 515 nm with streak camera time-resolved fluorescence upon excitation at 390 nm of Bts-Ag NPs_pABA_TiO_2_:Ru NPs in aqueous solution.

This observation contributes for the understanding of the increase in hydrogen evolution in the complete system, but leaves behind rationalization concerning the increase in methylene blue degradation comparatively to the unconnected system and even more so to the system prepared with Ag NPs with PVP termination. The most logical explanation for the rise in the photo-oxidation turnover is further increase in charge separation state lifetime, leading to the accumulation of reactive holes necessary for the multi-electron photo-oxidation process. The proposed hole stabilization mechanism is sensitive to Ag NPs termination, thus must involve the molecular structures on Ag NPs. A negative absorption signal was detected during the reaction with Bts-Ag NPs_pABA_TiO_2_:Ru NPs that is virtually absent for all the other materials tested (Fig. 6) and can be assigned to an emission resulting from a photophysical process occurring during the photocatalysis. The emission is centered at around 515 nm with a shoulder at 475 nm and is different from the emission of pure betalamic acid upon excitation at 405 nm (Fig. S8)^36^ but compatible with the photoluminesce profile of betanin submitted to alkaline hydrolysis (Fig. 6b). The lifetime of the emission emanated from the molecular structures on Ag NPs is 15.8 ps, estimated from time-resolved fluorescence (Fig. 6c). Poor light absorption of betanin at 405 nm and its low fluorescence quantum yield^37^ suggests that the direct excitation of surface betanin has little or no contribution to the emission observed during photocatalytic reaction. However, the molar attenuation coefficient of betalamic acid and neobetanin at 405 nm are higher than 20,000 L∙mol^−1^∙cm^−1^ and fluorescence of both compounds in solution is centered at around 500 nm^38^.

**Figure 7:**
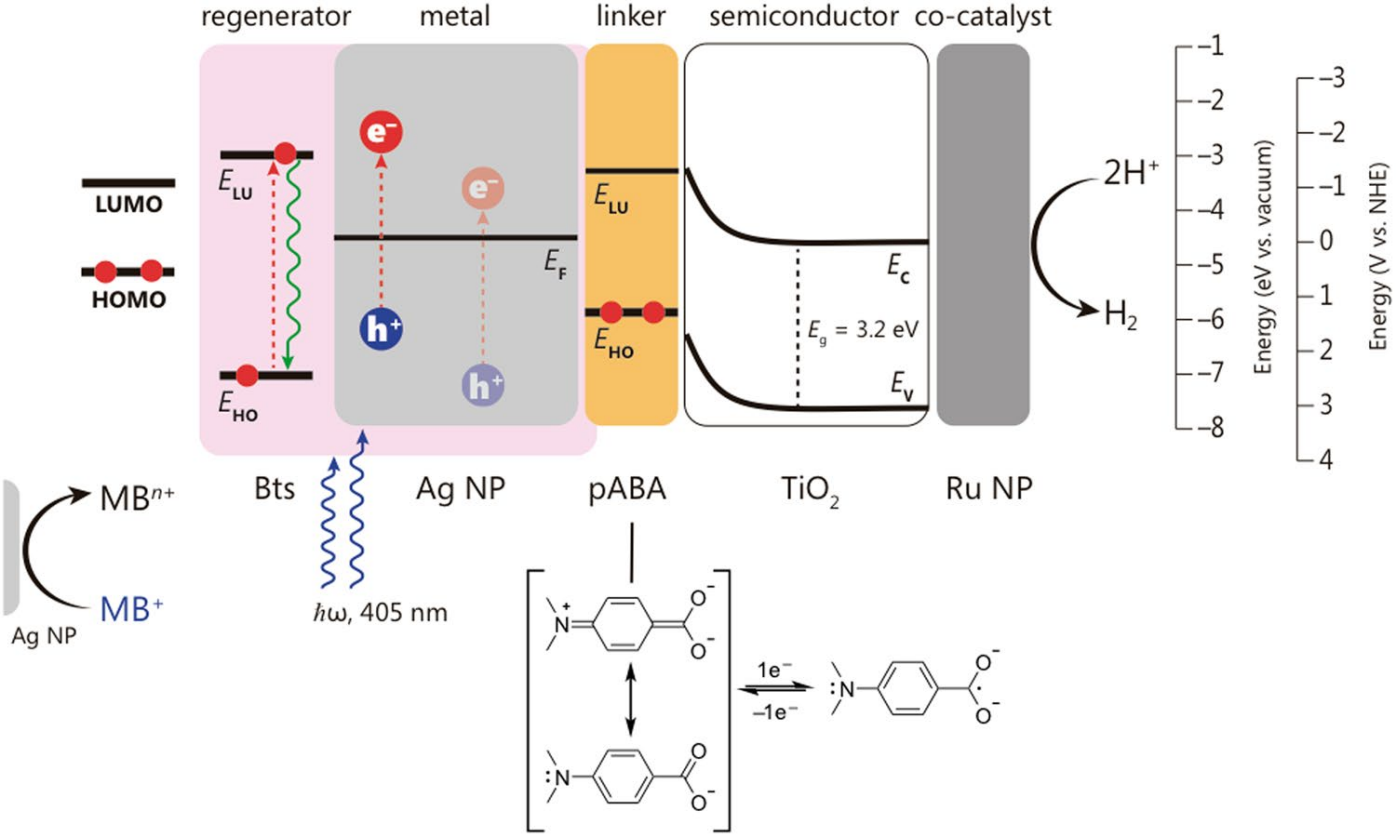
Charge particles pathways and reaction intermediates in the nano-hybrid assembly leading to a long-lived charge separated state. Electronic excitation is represented as dashed red arrows; this scheme does not necessarily represent the physical location where events take place. Energy levels were taken from the literature^39–43^.

One possible explanation for the stabilization of the hole and increase of electron-hole pair lifetime is that upon electron transfer from Ag NPs to TiO_2_, the Bts moieties transfer electrons to Ag NPs mediated by a proton release from Bts resulting in hole decay via a radiative reversible electron transfer, i.e. hole stabilization via proton-couple electron transfer, which is an essential process in chemical and biological catalysis, for example in water splitting, oxygen activation, proton reduction, nitrogen fixation, and ribonucleotide reductase reactions^44–46^. Another possibility is the concurrence between electron/hole recombination, electron transfer from Ag NPs to TiO_2_ and electron transfer from Ag NPs to the HOMO (highest occupied molecular orbital) of excited Bts. The latter process results in a stabilized anion-radical of Bts at the surface of the Ag NPs, increasing the persistence of holes. Next, electron transfer from Bts^−^ to h^+^-Ag NP is coupled to the radiative deactivation of the resulting excited state of Bts (Fig. 7). In both scenarios, the resulting accumulation of reactive holes in silver increases methylene blue abatement.

In conclusion, a silver based nano-hybrid assembly was designed combining natural pigments and molecular wire linkers used in DSSC and LSP-based photoconversion devices. The dramatic increase in the charge separation state lifetime compared to conventionally synthesize silver systems allowed the simultaneous production of hydrogen and oxidative pollutant abatement under visible light irradiation. This *bottom-up* modular architecture can be tuned to fit desired applications by changing the identity of a small number basic units, as well as the ratio between them. The plasmonic structure, the linker, the semiconductor and the metal co-catalyst can be permuted and combined in almost infinite ways, in strong parallel to metallic organic frameworks.

## Methods

### Materials synthesis

All reagents were purchased from commercial vendors (Sigma Aldrich) and used as received. Ag NPs (nanoparticles) decorated with betalamic acid were synthesized via base hydrolysis of betanin extracted from beetroot in an automated microfluidic reactor with an optimization algorithm that ensures the particles size and light absorption is homogeneous^25^. AgNO_3_ was used a metal precursor and betanin works as reducting and capping agent. The Ag NPs decorated with PVP were prepared with the same procedure and reactor but using PVP and NaBH_4_ as capping and reducing agent, respectively.

Ruthenium NPs on TiO_2_ (TiO_2__Ru, H_2_ evolution catalyst) was prepared by an impregnation-reduction method reported by Mishra *et al*.^47^. For the of Ru NPs on TiO_2_ with 3 nm nominal size, RuCl_3_· × H_2_O (0.02 M) was dissolved in dry ethanol (15 mL) in a round bottle flask with a mechanical stirrer and an argon inlet. The mixture was stirred at room temperature under argon for 24 h. Then, 0.2 M solution of NaBH_4_ in ethanol was added dropwise to the mixture under argon and continuously stirring. The solution turned dark grey within minutes but was left to stir for additional 24 h at room temperature to ensure complete reduction. Catalyst was recovered from solution after removing the solvent via centrifugation, and cleaned via a few cycles of washing/centrifugation with ethanol and water. The material was then dried at room temperature, yielding a grey powder. The TiO_2_ nanoparticles were supplied by Sachtleben, Hombikat UV 100 with a nominal size of 3 nm and anatase as crystal structure.

For the preparation of silver nano-hybrid architecture, Ag NPs decorated with betalamic acid or PVP (4.9 × 10^−3^ nM) were mixed with water solution of 4-aminobensoic acid (5 × 10^−6^ M) and stirred under argon for 15 min. TiO_2__Ru suspension in water (0.15 mg) was added afterwards. The elemental units were combined using a 1:1:1 ratio, i.e., each Ag NPs is linked via a single organic linker molecule to TiO_2__Ru.

### Standard characterization

SEM (scanning electron microscopy), DLS (dynamic light scatterring) and UV-Vis spectroscopy were used to monitor the Ag NPs homogeneity, average size and optical properties. UV-Vis spectra were acquired on a setup equipped with a deuterium-tungsten-halogen light source (DH-2000-BAL, Ocean Optics) and a USB4000 spectrometer (Ocean Optics). The size a distribution index of the particles was determined using DLS Malvern NanoS. SEM images of Ag NPs were acquired on a Leo 1550 SEM (Zeiss, Germany) instrument. The samples were supported on a silicon substrate and mounted in an aluminum stub. Prior to the measurements, samples were sputtered for 40 s with Au/Pd using a plasma current of 30 mA to minimize charging. Non-resonant Raman spectra were collected in a RENISHAW Raman microscope with excitation at 532 nm, objective of 20×, laser power at 100%, exposure time 10 s and five acquisitions. Liquid samples were mounted directly into a microscope glass slide.

### Photocatalytic studies

Ag NPs decorated with betalamic acid was prepared as described above. In the case of sample without linker, the set involving adding 4-aminobenzoic acid was replaced by simply adding an equivalent amount of pure water. Finally, methylene blue (1.3 × 10^−5^ M) and 1 drop of trifluoracetic acid were added to the reaction mixture. A schematic representation of the photocatalytic reactor is presented in Fig. S1. A 405 nm CW laser was used to excite selectively the Ag NPs LSP and the gaseous products were extracted via a carrier gas (argon) and analyzed in flow mode with a QMS (quadrupole mass-spectrometer), while methylene blue depletion and emission from the sample was monitored by UV-Vis spectroscopy in batch mode.

### Transient infrared absorption spectroscopy

Experiments were carried out in a femtosecond transient absorption spectrometer (Helios IR, Ultrafast Systems LLC) at room temperature. A one-box Ti:Sapphire based amplifier with integrated oscillator and pump lasers (800 nm, 40 fs, 3 kHz, Libra LHE, Coherent Inc.) was used to pump two TOPAS Primes coupled with frequency mixers (Light Conversion Ltd) to produce the depolarized visible excitation pulses ranging (405 nm) and the broad mid-IR probe spectrum. The samples were compacted between two CaF_2_ windows. Experiments were performed with humid samples, conferring conditions similar to what occurs during photocatalytic reaction. This approach also ensures good signal-to-noise ratio for pump-probe infrared measurements in transmission.

### Transient absorption spectroscopy

Experiments were carried out on laser based spectroscopy, with laser powers equating to less than one photon absorption per particle. Samples for transient absorption experiments were measured as suspensions in a liquid cell with quartz windows (1 mm). A Coherent Legend Ti:Sapphire amplifier (800 nm, 100 fs pulse length, 1 kHz repetition rate) was used. The output is split to pump and probe beams. Excitation pulses at the wavelength of 405 nm were acquired using an optical parametric amplifier (Topas C, Light Conversion). The probe pulses (a broad supercontinuum spectrum) were generated from the 800 nm pulses in a CaF_2_ crystal and split by a beam splitter into a probe pulse and a reference pulse. The probe pulse and the reference pulse were dispersed in a spectrograph and detected by a diode array. Instrumental response time is ~100 fs. Polarization of the pump was set at magic angle, 54.7° relative to the probe beam. The kinetic traces were extracted at 795 nm.

## Electronic supplementary material

Supplementary information
