# Supplementary material for: Nano-hybrid plasmonic photocatalyst for hydrogen production at 20% efficiency
Source: Sci Rep. 2017 Aug 17;7:8670. doi: 10.1038/s41598-017-09261-7 (PMC5561228; doi:10.1038/s41598-017-09261-7)
Supplement: Supplementary file 1 — Supplementary information [file 41598_2017_9261_MOESM1_ESM.doc]

# **Supplementary Information**

Nano-hybrid plasmonic photocatalyst for hydrogen production at 20% efficiency

Mariia V. Pavliuk,^1†^ Arthur B. Fernandes,^2†^ Mohamed Abdelah,^1,3†^ Daniel L. A. Fernandes,^1^ Caroline O. Machado,^2^ Igor Rocha,^4^ Yocefu Hattori,^1^ Cristina Paun,^1^
Erick L. Bastos,^2^* Jacinto Sá^1,5^*

^1^Departament of Chemistry-Ångström Laboratory, Uppsala University, 75120 Uppsala, Sweden

^2^Departament of Fundamental Chemistry, Institute of Chemistry, University of São Paulo, 05508-000 São Paulo, Brazil

^3^Department of Chemistry, Qena Faculty of Science, South Valley University, 83523 Qena, Egypt

^4^Departament of Engineering Sciences-Ångström Laboratory, Uppsala University, 75121 Uppsala, Sweden

^5^Institute of Physical Chemistry-Polish Academy of Sciences, 01-224 Warsaw, Poland

* jacinto.sa@kemi.uu.se; elbastos@iq.usp.br

† these authors contributed equally to the work

**Fig. S1.** Schematic representation of 4 mL quartz photoreactor used for the photocatalytic studies. Ag NPs LSP excited with 405 nm CW laser and products measure with QMS and fiber-optic UV-Vis spectroscopy.


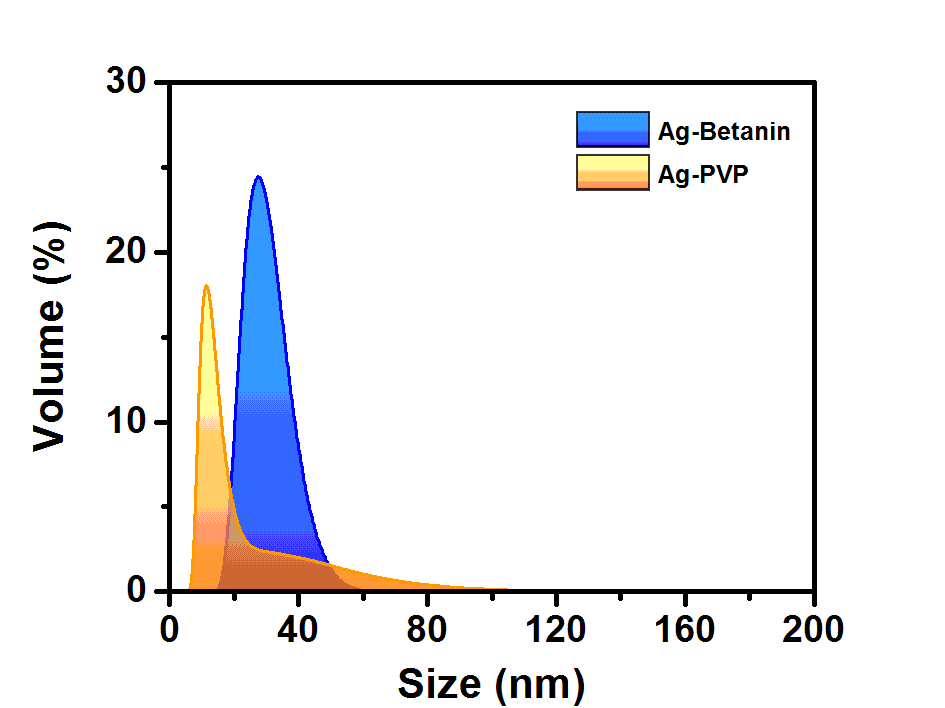


**Fig. S2**. Dynamic light scatteing (DLS) of silver NPs used.


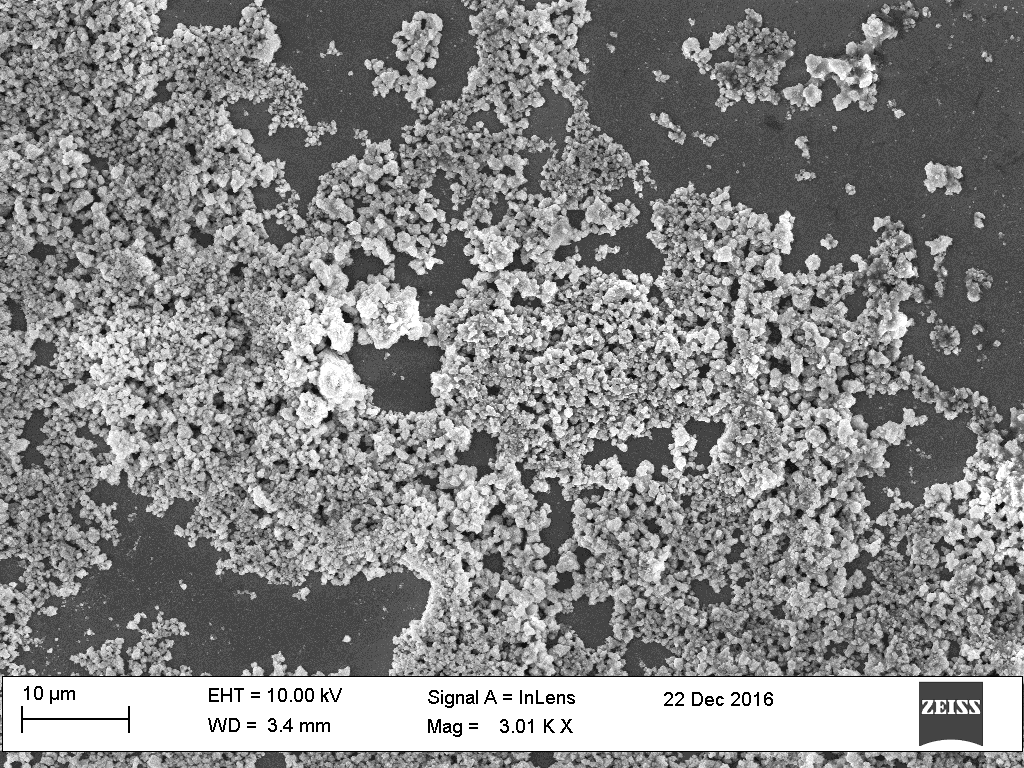


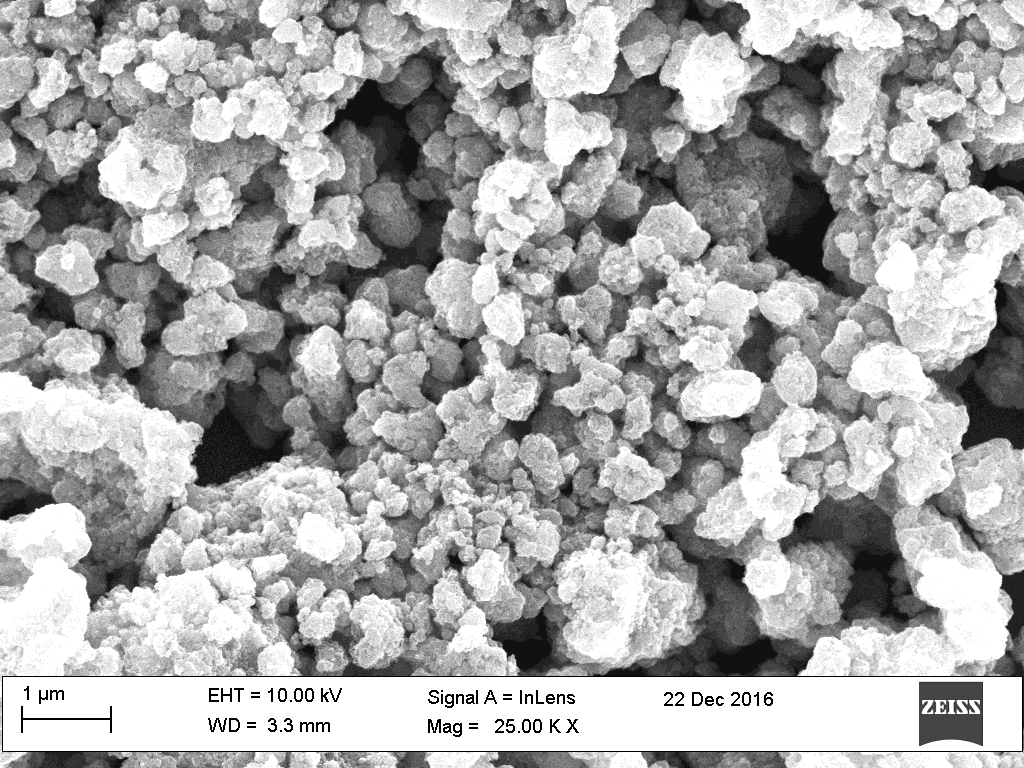


**Fig. S3**. Scanning electron microscopy images of silver NPs prepared with betanin extract.


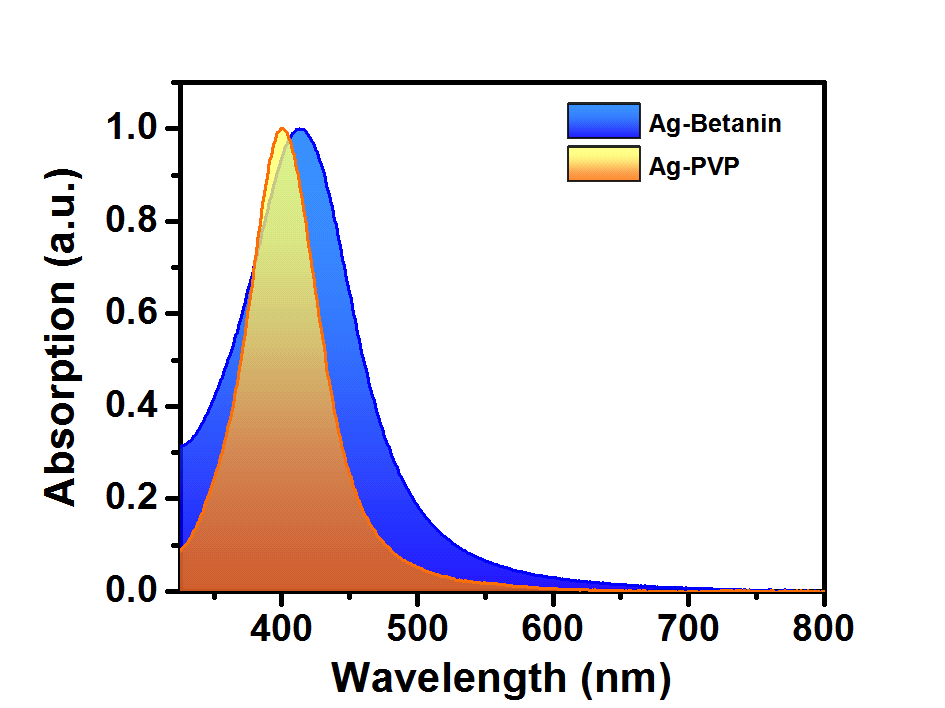


**Fig. S4**. UV-Vis absorption spectra of silver NPs used.


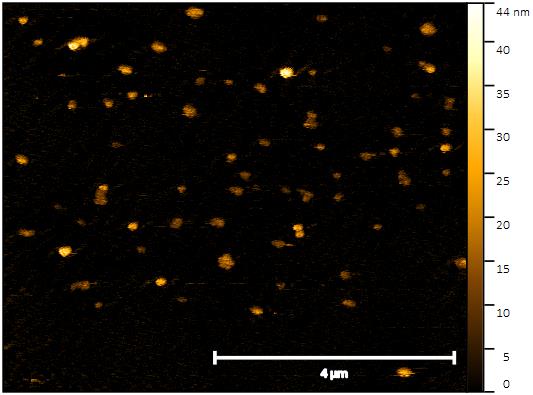


**Fig. S5**. Atomic force microscopy (AFM) image of silver NPs used.


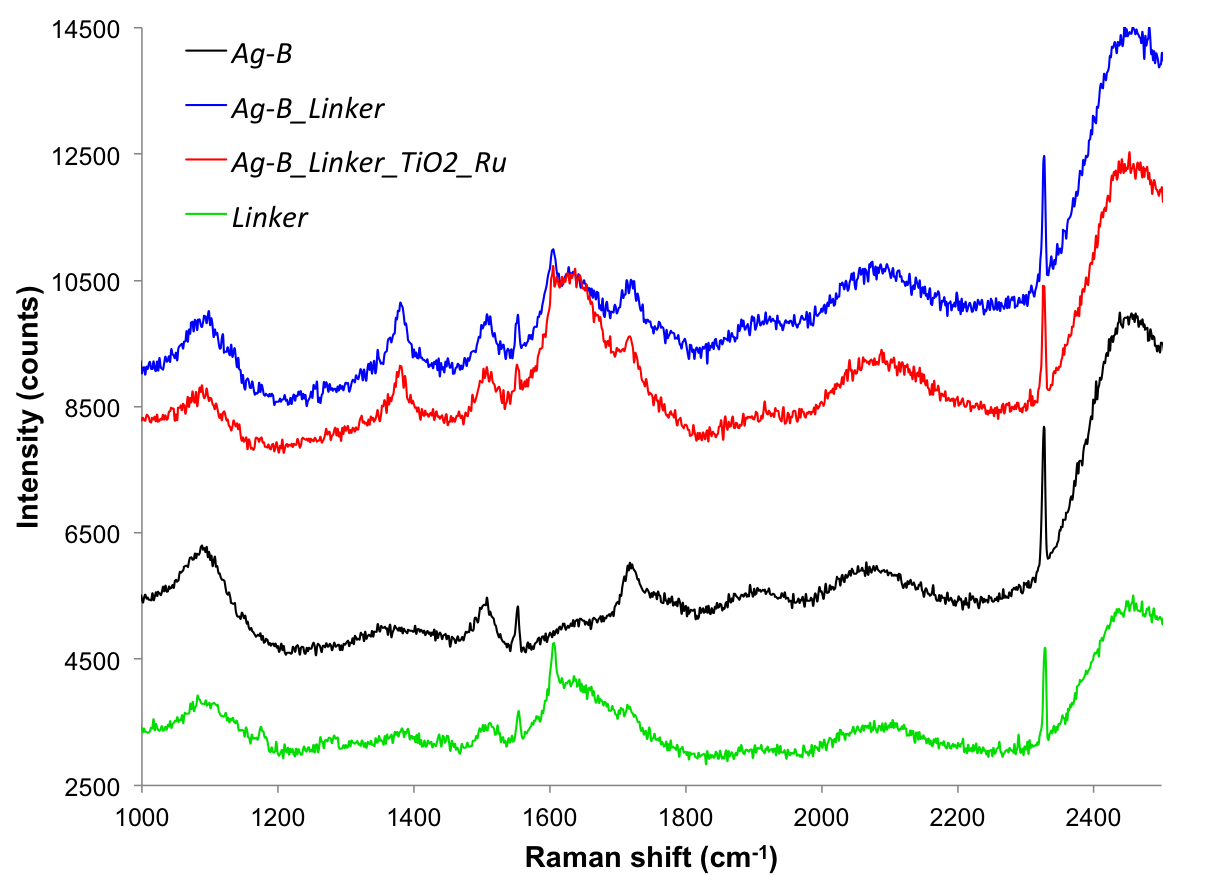


**Fig. S6**. Non-resonant Raman (excitation 532 nm) spectra showing the changes due to the sequential addition of the components of the silver based nano-hybrid architecture.


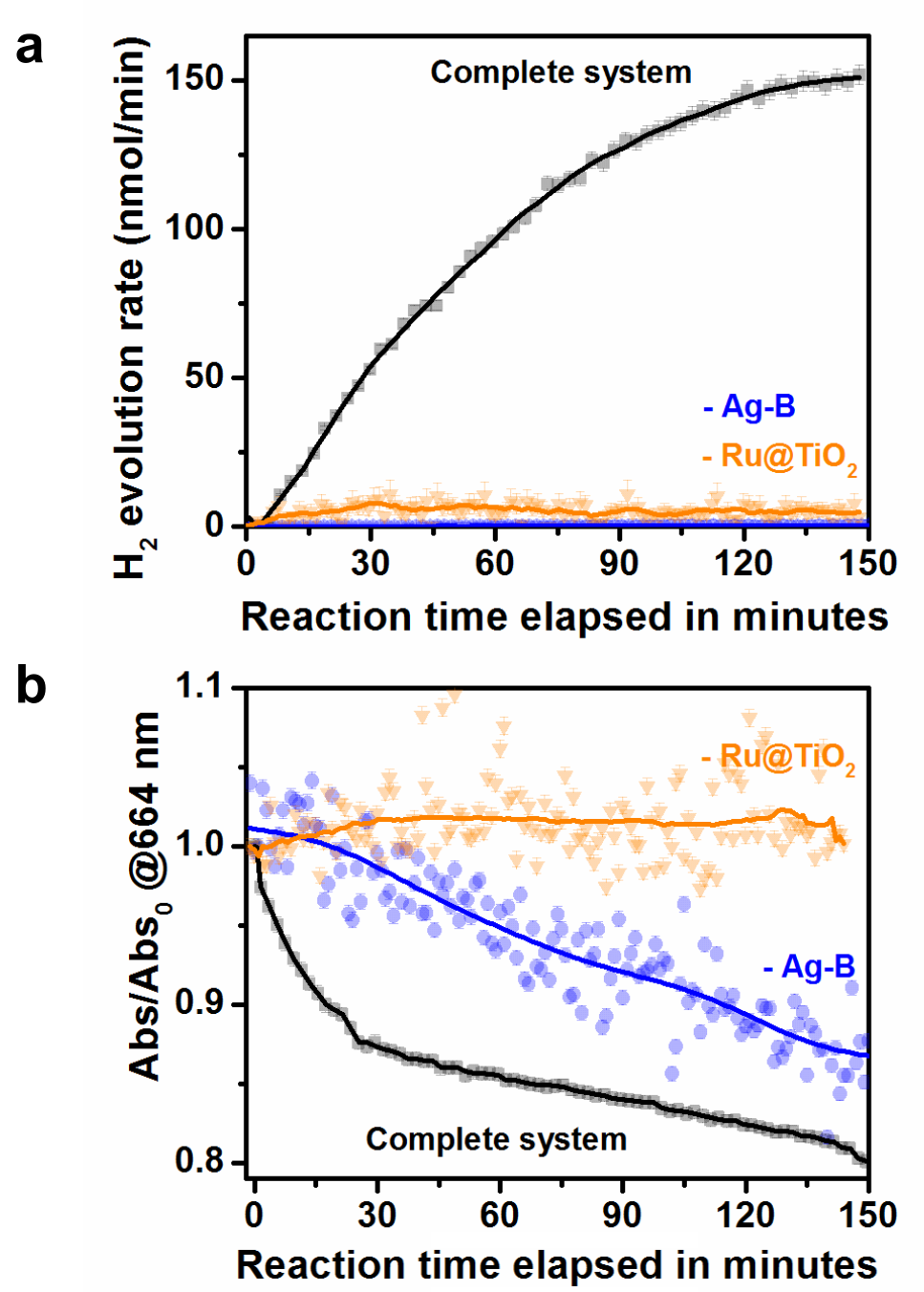


**Fig. S7**. Photo-catalytic control experiments with individual components. Complete silver based nano-hybrid assembly (black trace), only TiO_2_–Ru NPs (blue trace; –Ag-B) and only Bts-Ag NP (orange trace; –Ru@TiO_2_) only upon monochromatic excitation at 405 nm. A) H_2_ evolution; B) MB^+^ photo-oxidation.

**Fig. S8**. Steady-state fluorescence profile of (A) of betalamic acid in water (10 ppm AcOEt) and (b) betanin at pH = 12.
